# Draft genomes of *Klebsiella pneumoniae* and *Streptococcus anginosus* strains found in the urine of the same female patient

**DOI:** 10.1128/mra.01311-24

**Published:** 2025-03-06

**Authors:** Laricca Y. London, Chae Hee Lim, Jennifer L. Modliszewski, Nazema Y. Siddiqui, Tatyana A. Sysoeva

**Affiliations:** 1 Department of Biological Sciences, Alabama A&M University 6048 , Normal, Alabama, USA; 2 Department of Biological Sciences, The University of Alabama in Huntsville 14843 , Huntsville, Alabama, USA; 3 Duke University Center for Genomic and Computational Biology, Department of Bioinformatics and Biostatistics, Duke University 3065 , Durham, North Carolina, USA; 4 Division of Urogynecology and Reconstructive Pelvic Surgery, Department of Obstetrics and Gynecology, Duke University 571638 , Durham, North Carolina, USA; Rochester Institute of Technology, Rochester, New York, USA

**Keywords:** urinary microbiome, urinary tract infection, bladder commensals, recurrent UTI

## Abstract

Here, we report the draft genomes of *Klebsiella pneumoniae* 5008-1 and *Streptococcus anginosus* 5008-2 strains isolated from a catheterized urine sample obtained from an asymptomatic postmenopausal woman diagnosed with recurrent urinary tract infection and receiving vaginal estrogen cream.

## ANNOUNCEMENT

Since the early studies of microorganisms inhabiting the healthy urinary bladder of humans, there is an ongoing process in the identification of urinary commensal organisms and their interactions among themselves, with the host cells, and incoming pathogenic species (1). In this announcement, we describe the analysis of two bacterial strains*—K. pneumoniae* 5008-1 and *S. anginosus* 5008-2—isolated from an asymptomatic postmenopausal woman (58 years old) diagnosed with recurrent urinary tract infection. At the time of isolation, the patient was using vaginal estrogen cream, without prophylactic antibiotics. With patient consent and approval of the Duke University Medical Center Institutional Review Board (IRB, # Pro00083917), urine was obtained by aseptic catheterization and plated on sheep blood agar (2–5). Two types of colonies were observed and identified by matrix-assisted laser desorption ionization–time of flight (MALDI-TOF) mass spectrometry (6) as *K. pneumoniae* 5008-1 and *S. anginosus* 5008-2.

The isolates were propagated successfully in De-Man-Rogosa-Sharpe broth and stored as glycerol freezer stocks. Later, the strains were grown in tryptic soy media for obtaining biomass, and total DNA was extracted using the Qiagen DNEasy Blood and Tissue kit. Sequencing libraries were prepared with the Illumina DNA Prep kit. These libraries were sequenced on an Illumina NextSeq platform in 150 bp paired-end mode (Table 1). The raw paired-end reads were trimmed for adapter sequences and low-quality reads using the TrimGalore Toolkit (v.0.4.4) (7), which utilizes Cutadapt (v.1.16) (8). The quality of the raw reads was assessed using FastQC (v.0.11.7) (9). Three different assemblies were performed with the trimmed reads: (2) SPAdes (v.3.12.0) in default mode with the “careful” option enabled (1), SPAdes with the “careful” option enabled and a range of k-mers (35, 47, 59, 71, 83, 95, 107, 119, 127), and (3) Unicycler (v.0.4.4) in default mode (10). The final Unicycler assemblies were chosen based on their quality and completeness, as assessed by QUAST (v.4.5) (11) and BUSCO (v.3.0.2) (12) to identify conserved single-copy orthologs. To ensure no contamination, each assembly was run through the command line blastn (BLAST+, v.2.7.1) in megablast mode against the nt database (13). Default parameters were used here and throughout the analysis except where otherwise noted. The automated Prokaryotic Genome Annotation Pipeline v.3.1 was used to annotate obtained draft genomes before submitting to the databases, omitting contigs shorter than 200 bp (14).

**TABLE 1:** Genome assembly characteristics for two commensal urinary bacteria

Isolate	*K. pneumoniae* S5 5008-1	*S. anginosus* S6 5008-2
Draft genome assembly features		
No. of reads	31M	22.4M
No. of contigs	64	62
Contig N_50_	331, 358	121, 792
Coverage	216×	412×
Completeness (BUSCO)	99.9%	99.5%
Genome size	5,417,390 bp	2,001,769 bp
No. of total genes	5,352	2,074
GC content	56.8%	39.9%
Predicted markers of interest		
Plasmids	IncFIB(K), IncFII(K)	repUS43
CRISPR/Cas locus		1
Resistance	Tetracyclines, β-lactams, amphenicols	
Virulence genes	*6ccl, clpk1, fimH, mrkA, lutA, traT*	
Bacteriocins	Two colicins, bottromycin	Zoocin A

The analysis of the draft genomes of *K. pneumoniae* and *S. anginosus* for markers of interest was done using PlasmidFinder 2.1 (15, 16), Phast (17), Virulence Finder 2.0 (18, 19,20), ResFinder 4.0 (21, 22), CRISPRCasFinder (23), and Bagel4 (24). The results are presented in Table 1.

## Data Availability

This project was deposited at DDBJ/ENA/GenBank under the accession numbers JASENF000000000.1 and JASENE000000000.1 for *K. pneumoniae* S5 5008–1 and *S. anginosus* S6 5008–2 respectively. The raw Illumina sequencing data as sequence read archives are available as SRX20283061 and SRX20283062.
